# The Identification of *Streptococcus pasteurianus* Obtained from Six Regions in China by Multiplex PCR Assay and the Characteristics of Pathogenicity and Antimicrobial Resistance of This Zoonotic Pathogen

**DOI:** 10.3390/pathogens12040615

**Published:** 2023-04-18

**Authors:** Miaohang Ma, Shuoyue Wang, Xinchi Zhu, Xinchun Li, Yinli Bao, Xiang Chen, Zongfu Wu

**Affiliations:** 1 OIE Reference Lab for Swine Streptococcosis, College of Veterinary Medicine, Nanjing Agricultural University, Nanjing 210014, China; 2 Engineering Research Center for the Prevention and Control of Animal Original Zoonosis, College of Life Science, Longyan University, Longyan 364012, China; 3 Jiangsu Key Laboratory of Zoonosis, Yangzhou University, Yangzhou 225009, China

**Keywords:** *Streptococcus pasteurianus*, multiplex PCR, epidemiology, pathogenicity, antimicrobial resistance, zoonotic pathogen

## Abstract

*Streptococcus pasteurianus* is a zoonotic pathogen causing meningitis and bacteremia in animals and humans. A lack of accurate and convenient detection methods hinders preventing and controlling diseases caused by *S. pasteurianus*. Additionally, there is limited knowledge about its pathogenicity and antimicrobial resistance characteristics, as there are only three complete genome sequences available. In this study, we established a multiplex PCR assay for the detection of *S. pasteurianus*, which was applied to six fecal samples from cattle with diarrhea and 285 samples from healthy pigs. Out of the samples tested, 24 were positive, including 5 from pig tonsils, 18 from pig hilar lymph nodes, and 1 from cattle feces. Two strains were isolated from positive samples, and their complete genomes were sequenced. The two strains were non-virulent in mice and multidrug-resistant by the antimicrobial susceptibility test. We first found the presence of genes *tet(O/W/32/O)* and *lsa(E)* in *S. pasteurianus*, leading to resistance to lincosamides and tetracyclines. The convenient and specific multiplex PCR assay provides essential technical support for epidemiological research, and the complete genome sequence of two non-virulent strains contributes to understanding this zoonotic bacterium’s genomic characteristics and pathogenesis.

## 1. Introduction

*Streptococcus pasteurianus* was classified as *Streptococcus bovis* biotype II/2 [1,2] and later classified as a new species, *S. pasteurianus*, by phylogenetic tree analysis of gene s*odA* encoding manganese-dependent superoxide dismutase [3]. It is considered to be a zoonotic pathogen, causing urinary tract infection [4], endocarditis [5], meningitis [6,7], bacteremia [8,9], sepsis [9,10], and other symptoms [11,12,13,14] and even death in neonates, adults, the elderly, and immunocompromised patients. Furthermore, it may be associated with human gastrointestinal malignancy [15]. To date, 37 papers have reported cases of human infection caused by *S. pasteurianus*, occurring in 15 countries, namely 10 in America, 8 in Japan, 5 in China, 2 in Spain, 2 in France, and 1 in Argentina, Australia, Britain, Costa Rica, Korea, the Netherlands, Portugal, Thailand, Turkey, and India (further details provided in Table S1). Additionally, geese [16,17], ducks [18], turkeys [19], cattle [20], and emperor tamarin [21] are susceptible to *S. pasteurianus* infection, which can result in septicemia, meningitis, and other symptoms with a high mortality rate. Our group was the first to confirm that this bacterium can cause meningitis in pigs and is a new pathogen of swine streptococcosis [22]. Animal infections involve 6 species across 6 countries, spanning America, Austria, Brazil, Britain, China, and Italy (further details provided in Table S2). Currently, there are no available molecular typing and serotyping methods for *S. pasteurianus*. The transmission of this bacterium in animals remains uncertain. However, in humans, neonatal infections have been reported, which are likely caused by vertical transmission [23] and horizontal transmission [14,24]. A recent study indicated that a cluster of neonatal sepsis was caused by *S. pasteurianus*, possibly due to fecal–oral or contact transmission [25]. Overall, there are no clear reports on the mode of transmission of *S. pasteurianus*, nor are there any established management practices to reduce the risk of infection.

Due to its potential to cause severe infections in both humans and animals, there is a critical need to develop an accurate, rapid, and convenient detection method for *S. pasteurianus*. Biochemical tests are common methods to identify *S. pasteurianus* [7,9]. However, the biochemical reaction of bacteria from *Streptococcus* genus is active with significant differences between strains, and the phenotype of the biochemical reaction is unstable [26]. So, only the biochemical test is not accurate enough to identify *S. pasteurianus*. In 2002, Poyart et al. proposed that the *sodA* sequence can be used to identify *S. pasteurianus*, and *sodA* sequence identity (from positions 25 to 510) of *S. pasteurianus* strains should be ≥98.9% with that of type strain NCTC 13784 [3]. Although it is an accurate method for identifying *S. pasteurianus*, detecting a large number of clinical samples based on this method is inconvenient and time-consuming. In 2017, Hatrongjit et al. established a PCR assay for *S. pasteurianus* using the *SGPB0680* gene encoding a cell wall surface protein as a species-specific gene [27]. However, our previous research found that some strains that contained this gene were not *S. pasteurianus*, indicating that this gene cannot accurately distinguish *S. pasteurianus* from its related species. So far, it is infeasible to identify *S. pasteurianus* based on one species-specific gene by PCR method. Thus, in addition to gene *SGPB0680* (*E8M05_RS04035* in strain WUSP067), two more genes, *E8M05_RS05155* encoding a major facilitator superfamily (MFS) transporter and *E8M05_RS06300* encoding a carboxylesterase family protein, were included, and strains with the presence of three genes were identified as *S. pasteurianus* [22]. However, the rapid and convenient detection method based on these three genes was not established, and no *S. pasteurianus* strain was isolated from pig tonsils or hilar lymph nodes in our previous study [22].

In this study, we established a multiplex PCR assay for this bacterium based on these three genes, evaluated the sensitivity and specificity of this assay, and applied the assay to detect *S. pasteurianus* from pig mesenteric lymph nodes, hilar lymph nodes, tonsils, and cattle feces. In addition, two *S. pasteurianus* strains were isolated from the above samples and sequenced, and the characteristics of the pathogenicity and antimicrobial resistance of these two strains were investigated.

## 2. Materials and Methods

### 2.1. Bacterial Strains and Culture Conditions

Information on strains is shown in Table 1. The *S. pasteurianus*, *Streptococcus suis*, *Streptococcus equi* subsp. *zooepidemicus*, *Streptococcus agalactiae*, *Streptococcus pluranimalium*, *Enterococcus* sp., and *Streptococcus hyovaginalis* were grown in Todd–Hewitt broth (THB, Hope, Qingdao, China) or agar medium at 37 °C. *Escherichia coli* was grown in Luria–Bertani medium (LB, Hope, Qingdao, China) at 37 °C. The *Bacillus subtilis* was grown in nutrient broth (NB, Hope, Qingdao, China) or agar medium at 30 °C. The *Aeromonas hydrophila* was grown in tryptic soy broth (TSB, Hope, Qingdao, China) or agar medium at 37 °C. The *Klebsiella pneumoniae* was grown in MacConkey agar (Hope, Qingdao, China) at 37 °C.

### 2.2. DNA Extraction

The Bacterial DNA Kit (TIANGEN, Beijing, China) was used to extract the bacterial genome, following the manufacturer’s guidelines. The DS-11^+^ Spectrophotometer (DeNovix Inc, Wilmington, DE, USA) was used to quantify DNA.

### 2.3. Multiplex PCR Assay

Primers for the multiplex PCR assay are shown in Table 2. The single PCR amplification was performed in 25 μL reactions, including 12.5 μL of 2 × Rapid Taq Master Mix (Vazyme, Nanjing, China), 1 μL of each primer, 9.5 μL of ddH_2_O, and 1 μL of the template, by an initial 3 min denaturation at 95 °C, followed by 30 cycles of 15 s at 95 °C, 15 s at 55 °C, and 12 s extension at 72 °C, with a final extension step of 5 min at 72 °C. Based on single PCR amplification conditions, the multiplex PCR assay was optimized by varying the ratio of each primer pair and the annealing temperature. The optimal reaction system is 12.5 μL of 2 × Rapid Taq Master Mix, 2.5 μL of 1-F/R, 0.5 μL of 2-F/R, 1.0 μL of 3-F/R, 3.5 μL of ddH_2_O, and 1 μL of the template. The optimal annealing temperature is 52.0 °C. The amplification product was analyzed on 1.5% agarose (Tsingke, Beijing, China) in 1 × TAE buffer, stained with GelstainRed (BioScience, Shanghai, China), and shown by Gel Doc XR+ (Bio-Rad, Hercules, CA, USA).

### 2.4. Sensitivity and Specificity Assays

Sensitivity and specificity tests were conducted to evaluate the performance of the multiplex PCR assay. For the sensitivity assay, bacteria from 5.46 × 10^5^ to 5.46 × 10^1^ colonies forming unit (CFU), and DNA from 17.137 to 1.71376 × 10^−4^ ng, were used as templates for the multiplex PCR assay. For the specificity assay, eleven different bacteria, listed in Table 1, were used as templates, consisting of *S. suis*, and *S. equi* subsp*. zooepidemicus*, *S. agalactiae*, *S. pluranimalium*, *B. subtilis*, *A. hydrophila*, *K. pneumoniae*, *S. dysgalactiae*, *E. coli*, *Enterococcus* sp., and *S. hyovaginalis*.

### 2.5. Sample Processing and S. pasteurianus Isolation

The multiplex PCR assay was performed on six fecal samples obtained from cattle with diarrhea in Inner Mongolia. Moreover, the assay was used to detect the presence of *S. pasteurianus* in 285 samples obtained from healthy pigs. These samples comprised 50 tonsils from Chongqing, 50 hilar lymph nodes from Yunnan, 50 mesenteric lymph nodes from Jiangsu, 45 hilar lymph nodes and 30 tonsils from Guangxi, as well as 60 tonsils from Sichuan (Table 3). The cattle feces were transferred to THB containing 15 mg/L polymyxin (Macklin, Shanghai, China) and 30 mg/L nalidixic acid (Macklin, Shanghai, China). A 0.1 g tissue sample was taken from healthy pigs’ tonsils or lymph nodes. These samples were homogenized in FastPrep 5G (MP Biomedicals, Santa Anna, CA, USA) with 900 μL of 1 × PBS. The homogenate was transferred to THB containing 15 mg/L polymyxin and 30 mg/L nalidixic acid in a 1:50 ratio. All samples were cultured for 8–10 h at 37 °C and 5% CO_2_. To collect bacteria, 2–4 mL of culture was taken and centrifuged at 5000 rpm for 10 min. The genome DNA extracted from the bacteria was used as the template for multiplex PCR assay. The *sodA* sequence analysis was performed on positive DNA samples to validate the multiplex PCR results. Positive samples were streaked on THB agar plates containing polymyxin (15 mg/L) and nalidixic acid (30 mg/L). *S. pasteurianus* isolates from positive samples were identified by selecting 100 colonies from each agar plate.

### 2.6. Sequencing, Assembly, Annotation, and Bioinformatics Analysis of the Genome

Sequencing, assembly, annotation, and bioinformatics analysis of the bacterial genome are essential steps in understanding the genetic characteristics of a bacterial species. The complete genome sequencing for strains WUSP070 and WUSP074 was performed by Benagen (Wuhan, China) by joining the third-generation Nanopore and second-generation Illumina technologies. Illumina sequencing by NovaSeq 6000 PE150 (Illumina, San Diego, CA, USA) generated 1,113,403,821 bp (WUSP070) and 1,329,269,684 bp (WUSP074) clean data. Nanopore sequencing by PromethION (Oxford Nanopore Technologies, Oxford, UK) generated 2,460,408,362 bp (WUSP070) and 3,601,438,875 bp (WUSP074) clean data. Unicycler [28] software (version 0.5.0) was used to assemble the genome. Prokka [29] software (Version 1.14.6) was used to annotate the genome. The sequences and annotations were deposited in NCBI (Accessions Nos. NZ_CP116957.1 and NZ_CP116958.1). ResFinder 4.0 [30] was used to analyze antibiotic resistance genes. The integrative conjugative element (ICE) was predicted by VRprofile2 [31]. The Virulence Factor Database (VFDB), available at http://www.mgc.ac.cn/VFs/ (accessed on 15 October 2022), was utilized to predict virulence factors, with a cut-off value of 80% amino acid identity and 90% coverage.

### 2.7. Antimicrobial Susceptibility Testing

Antimicrobial susceptibility testing is essential for understanding the antimicrobial resistance characteristics of a bacterium. The minimum inhibitory concentrations (MICs) of antimicrobials tested on *S. pasteurianus* isolates were determined using the broth microdilution method and interpreted according to the Clinical and Laboratory Standards Institute (CLSI, 2020) guidelines. *S. aureus* ATCC 29213 was used as a quality control strain. The breakpoints for resistance to antimicrobials tested were adopted according to our previous research [22].

### 2.8. Mice Infection 

Mice infection experiments are essential for understanding the bacterial pathogenesis. Three-week-old newly weaned specific pathogen-free (SPF) ICR mice (SPF, Beijing, China) were used as the infection model according to our previous study [22]. *S. pasteurianus* was injected intraperitoneally into mice (10 per group) at a dose of 1.5 × 10^8^ cfu per mouse. As a negative control, 5 mice were infected with PBS. Mortality was monitored for 2 weeks post-infection. The Log-rank (Mantel-Cox) test was used to analyze the result of animal infection.

## 3. Results

### 3.1. Multiplex PCR Assay for S. pasteurianus

**Figure 1:**
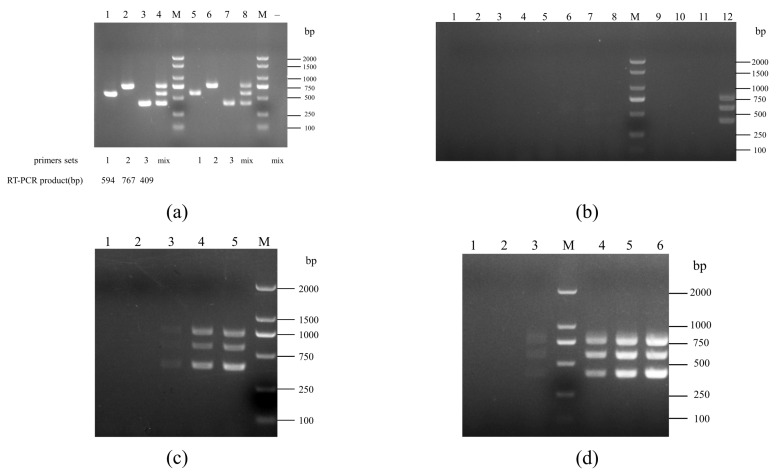
Multiplex PCR Assay for *S. pasteurianus*. (**a**) Single and multiplex PCR amplification. Lanes 1–4: templates, the genome of WUSP067; primers, 1–F/R, 2–F/R, 3–F/R and a mixture of three primer pairs. Lanes 5–8: templates, culture of WUSP067; primers, 1–F/R, 2–F/R, 3–F/R and a mixture of three primer pairs. M, DNA Marker; – negative control, H_2_O. (**b**) Specificity of multiplex PCR. The culture of bacteria as templates: 1, *S. equi* subsp. *zooepidemicus* strain ATCC 35246; 2, *S. agalactiae* strain GD201008–001; 3, *S. pluranimalium* strain ML20171221B6–2; 4, *B. subtilis* strain 1.460; 5, *A. hydrophila* isolate WUQT018; 6, *K. pneumoniae* isolate WUQT019; 7, *S. dysgalactiae* isolate WUQT020; 8, *E. coli* isolate WUQT022; 9, *Enterococcus* sp isolate WUQT024; 10, *S. hyovaginalis* isolate WUQT033; 11, *S. suis* strain SC070731; 12, *S. pasteurianus* strain WUSP067. (**c**) Sensitivity of multiplex PCR. The cfu of WUSP067,1–5: 5.46 × 10^1^ cfu, 5.46 × 10^2^ cfu, 5.46 × 10^3^ cfu, 5.46 × 10^4^ cfu, and 5.46 × 10^5^ cfu. (**d**) Sensitivity of multiplex PCR. The DNA of WUSP067, 1–6: 171.37 fg, 1.7137 pg, 17.137 pg, 171.37 pg, 1.7137 ng, and 17.137 ng.

As shown in Figure 1a, the fragments obtained by the single PCR amplification for genes *E8M05_RS04035*, *E8M05_RS05155*, and *E8M05_RS06300*, are 594, 767, and 409 bp, respectively, using the DNA or the culture of *S. pasteurianus* strain WUSP067 as templates; after optimizing reaction conditions, the multiplex PCR allowed amplification of three fragments simultaneously from the DNA or the culture of strain WUSP067. Only the culture of *S. pasteurianus* can amplify three fragments by this multiplex PCR, not the rest of the 11 other bacteria (Figure 1b). The sensitivity assay was performed with 10-fold dilutions of *S. pasteurianus* strain WUSP067 culture and DNA. The detection limit of the multiplex PCR assay was 5.46 × 10^3^ cfu (Figure 1c), and 17.137 pg DNA (Figure 1d). Therefore, we have developed a multiplex PCR suitable for *S. pasteurianus* identification. 

### 3.2. Application of the Multiplex PCR Assay

The multiplex PCR assay was applied to detect *S. pasteurianus* from six fecal samples collected from cattle with diarrhea, and one sample was positive for *S. pasteurianus* (Table 3). In addition, 140 tonsils, 95 hilar lymph nodes, and 50 mesenteric lymph nodes from healthy pigs from different provinces were detected by this assay. The results showed that the positive rate of pig tonsils was 3.57% (5/140), the positive rate of pig hilar lymph nodes was 18.95% (18/95), and *S. pasteurianus* was not detected in pig mesenteric lymph nodes (Table 3). Among the 285 samples from healthy pigs, the positive rate of *S. pasteurianus* in the hilar lymph nodes was the highest, followed by the tonsils, and the lowest in the mesenteric lymph nodes. 

To further validate this assay, *sodA* was amplified from these 24 *S. pasteurianus* positive samples. Sequence analysis showed that the sequence identity of *sodA* (from positions 25 to 510) obtained from 24 positive samples was all > 98.9% with that of type strain NCTC 13784 (Table S3). In addition, two *S. pasteurianus* strains WUSP070 and WUSP074 were isolated from these positive samples, and the information on the two strains is shown in Table 1.

### 3.3. The Complete Genome Sequence of Two S. pasteurianus Strains

The genome of strain WUSP070 comprises a single circular chromosome of 2,290,055 bp with G+C contents of 37.40%, 6 rRNA operons, 70 tRNA genes, and 2196 CDSs predicted in the chromosome. The genome of strain WUSP074 comprises a single circular chromosome of 2,371,672 bp with G+C contents of 37.34%, 6 rRNA operons, 70 tRNA genes, and 2309 CDSs predicted in the chromosome. No plasmid is present in the two strains.

### 3.4. The Characteristics of Antimicrobial Resistance of Two S. pasteurianus Strains

**Figure 2:**
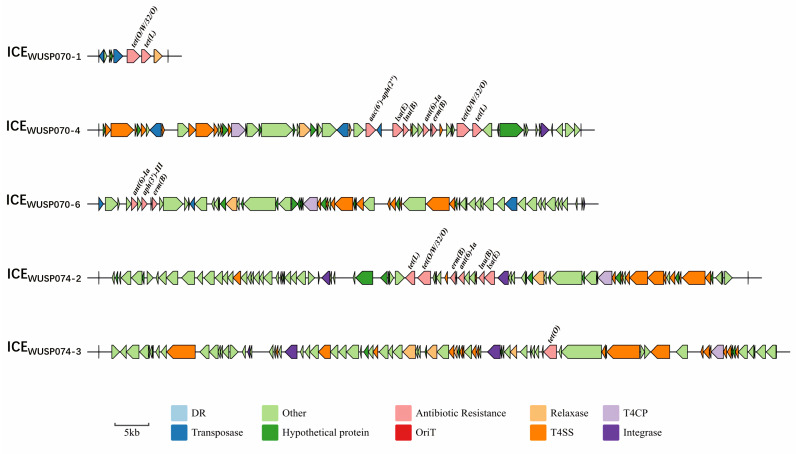
Vehicles for harboring antimicrobial resistance genes in *S. pasteurianus*. The ICE_WUSP070-1_ contained genes *tet(O/W/32/O)* and *tet(L)*. The ICE_WUSP070-4_ contained genes *acc(6′)-aph(2″)*, *lsa(E)*, *lnu(B)*, *ant(6)-Ia*, *erm(B)*, *tet(O/W/32/O)*, and *tet(L)*. The ICE_WUSP070-6_ contained genes *ant(6)-Ia*, *aph(3′)-III*, and *ermB*. The ICE_WUSP074-2_ contained genes *tet(L)*, *tet(O/W/32/O)*, *erm(B)*, *ant(6)-Ia*, *lnu(B)*, and *lsa(E).* The ICE_WUSP074-3_ contained gene *tet(O)*.

The presence of antimicrobial resistance genes *erm(B)*, *lnu(B)*, *tet(O/W/32/O)*, *tet(L)*, *aac(6′)-aph(2″)*, and *lsa(E)* in strain WUSP070 contributed to its resistance to erythromycin, lincomycin, clindamycin, doxycycline, and gentamycin (Table 4). The presence of antimicrobial resistance genes *erm(B)*, *lnu(B)*, *lsa(E)*, *tet(O)*, *tet(O/W/32/O)*, and *tet(L)* in strain WUSP074 led to resistance to erythromycin, lincomycin, clindamycin, and doxycycline (Table 4). The strain resistant to three or more classes of antimicrobial agents is considered to be multidrug-resistant [32]. Thus, strains WUSP070 and WUSP074 are multidrug-resistant. To explore vehicles harboring antimicrobial resistance genes, the ICEs were predicted. Six ICEs were predicted in strain WUSP070, and three contained antimicrobial resistance genes. The ICE_WUSP070-1_ (from *M0P24_RS00080* to *M0P24_RS00115*) harboring a 23-bp *att* sequence 5′-ggttctgttgcaaagttttaaat-3′ in the flanking region contained genes *tet(O/W/32/O)* and *tet(L)* (Figure 2). The ICE_WUSP070-4_ (from *M0P24_RS04110* to *M0P24_RS04435*) harboring a 16-bp *att* sequence 5′-tactgttttaacaatg-3′ in the flanking region contained genes *aac(6′)-aph(2″)*, *lsa(E)*, *lnu(B)*, *ant(6)-Ia*, *erm(B)*, *tet(O/W/32/O)*, and *tet(L)* (Figure 2). The ICE_WUSP070-6_ (from *M0P24_RS10640* to *M0P24_RS10985*) contained genes *ant(6)-Ia*, *aph(3′)-III*, and *ermB* (Figure 2). Three ICEs were predicted in strain WUSP074, and the ICE_WUSP074-2_ (from *M0P24_RS06290* to *M0P24_RS06755*) harboring a 15-bp *att* sequence 5′-tttttgaagttctgg-3′ in the flanking region contained genes *tet(L)*, *tet(O/W/32/O)*, *erm(B)*, *ant(6)-Ia*, *lnu(B),* and *lsa(E).* The ICE_WUSP074-3_ (from *M0P24_RS07560* to *M0P24_RS08050*) harboring a 15-bp *att* sequence 5′-aatatcaaaaatcag-3′ in the flanking region contained gene *tet(O)* (Figure 2). These ICEs were not present in a virulent strain WUSP067 isolated from a diseased pig from our previous study [22].

### 3.5. The Pathogenicity Characteristics of Two S. pasteurianus Strains

**Figure 3:**
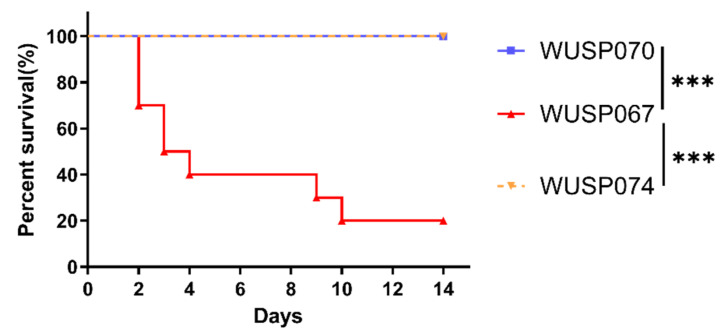
The survival curve of mice. Mice (10 per group) were injected with *S. pasteurianus* strains (WUSP067, WUSP070, and WUSP074) at a dose of 1.5 × 10^8^ cfu per mouse. For two weeks after infection, mortality was tracked. To analyze the results, the Log-rank (Mantel-Cox) test was used. ‘***’ indicates *p* < 0.001.

Table S4 reveals that strains WUSP070 and WUSP074 have 13 and 12 predicted virulence factors, respectively. However, on the 14th day post-infection, mice infected with strains WUSP070 and WUSP074 displayed a 100% survival rate, while mice infected with WUSP067 exhibited only a 20% survival rate (Figure 3). This observation confirms that strain WUSP067 is more pathogenic than strains WUSP070 and WUSP074.

## 4. Discussion

As indicated in Tables S1 and S2, there have been 44 papers reporting cases of *S. pasteurianus* infection in both humans and animals. Among them, 27 papers (61.36%) were published in the last decade, indicating a recent increase in attention towards *S. pasteurianus*. We first confirmed that this bacterium is a new pathogen of swine streptococcosis, and it can cause meningitis in pigs. In addition, *S. pasteurianus* was detected in pig tonsils, pulmonary hilar lymph nodes, and gut [22,33,34]. We proposed that healthy pigs’ tonsils and pulmonary hilar lymph nodes may be reservoirs of this bacterium [22]. So far, there are only three complete genome sequences of this bacterium, two human strains (ATCC 43144 and NCTC 13784) and a virulent strain WUSP067 isolated from a pig. The complete genome sequence of two non-virulent strains WUSP070 and WUSP074 provided in this study contributes to understanding the genomic characteristics of this zoonotic bacterium and identifying virulence factors by comparative genomic analysis. 

So far, the identification of *S. pasteurianus* is mainly by biochemical testing systems, PCR assay based on *SGPB0680* gene, and *sodA* gene sequence analysis. As mentioned above, those methods have shortcomings. In this study, we established a multiplex PCR assay to conveniently and accurately identify *S. pasteurianus*. The detection limit was 17.137 pg using bacterial DNA as templates. After optimization, this assay can also be used to detect bacterial cultures directly with the detection limit of 5.46 × 10^3^ cfu. Using this assay, 24 samples were positive for *S. pasteurianus*, including pig hilar lymph nodes, tonsils, and cattle feces. Furthermore, *sodA* sequence (from positions 25 to 510) analysis further confirmed that these 24 samples contained *S. pasteurianus*. However, this method should be further optimized in the future to improve the sensitivity of bacterial cultures. In our previous study, the detection rate of *S. pasteurianus* in pig tonsils was lower than that in pig hilar lymph nodes [22], and we wondered if it was related to tissues or regions where samples were collected. In this study, we found that the detection rate of *S. pasteurianus* in pig hilar lymph nodes was significantly different in different regions. For example, there was a 36% detection rate in Yunnan province, while none of the pig hilar lymph nodes was positive in Guangxi province. Thus, the detection rate of *S. pasteurianus* in pig tissues may be more related to regions where samples were collected, not tissues.

The virulence of bacteria such as *S. suis* cannot be purely defined by the source of the isolated strain [35]. Some *S. suis* strains isolated from healthy pigs are pathogenic and a source of infection for susceptible pigs and humans [36]. In this study, we isolated *S. pasteurianus* strain WUSP074 from a healthy pig tonsil, and mice infection experiments revealed that the strain was non-pathogenic. Whether, like *S. suis*, some tonsillar-derived *S. pasteurianus* strains are pathogenic and serve as a source of infection warrants further study. In addition, strain WUSP070 was isolated from cattle feces with diarrhea. Trotta et al. isolated two strains from calves with neurological hyperacute symptoms in 2019 [20]. If *S. pasteurianus* can cause cattle diarrhea needs further research. 

For *S. pasteurianus*, the information on antimicrobial resistance and the spread of antibiotic resistance genes is very few. Most strains are resistant to macrolides, lincosamides, and tetracyclines [22,26,27]. In this study, two isolates WUSP070 and WUSP074 are also resistant to macrolides, lincosamides, and tetracyclines, and we first reported the presence of genes *tet(O/W/32/O)* and *lsa(E)* in *S. pasteurianus* that contribute to their resistance. In a duckling isolate AL101002, gene *erm(B)* was located at the Tn916-like element, and genes *tet(M)*, *tet(L)*, and *erm(T)* were located at the Tn916-IS1216 cluster [37]. In strain WUSP067, genes *erm(B)* and *acc(6′)-aph(2″)* were located at ICE_WUSP067-1_; genes *tet(M)* and *tet(L)* were located at ICE_WUSP067-2_ [22]. In this study, two isolates WUSP070 and WUSP074 have different vehicles, five ICEs, harboring antimicrobial resistance genes (Figure 2). Thus, ICEs may be the main vehicle for the spread of antibiotic-resistance genes in *S. pasteurianus.*

## 5. Conclusions

In this study, we established a convenient and specific multiplex PCR assay suitable for *S. pasteurianus* identification. This assay can be used to detect a large number of clinical samples, which provides essential technical support for the epidemiological research of *S. pasteurianus*. We first reported the presence of genes *tet(O/W/32/O)* and *lsa(E)* in *S. pasteurianus*, which leads to its resistance to lincosamides and tetracyclines. The complete genome sequence of two non-virulent strains contributes to understanding this zoonotic bacterium’s genomic characteristics and pathogenesis.

## Figures and Tables

**Table 1:** The information of strains or isolates used in this study.

Strain	Species	Origin	NCBI Accession
WUSP067	*S. pasteurianus*	isolated from a newly weaned piglet’s brain with meningitis	NZ_CP039457
WUSP070	*S. pasteurianus*	isolated from a diarrheal cattle fecal sample	NZ_CP116957.1
WUSP074	*S. pasteurianus*	isolated from a healthy porcine tonsil	NZ_CP116958.1
SC070731	*S. suis*	isolated from a pig with meningitis	NC_020526
ATCC 35246	*S. equi* subsp. *zooepidemicus*	isolated from a dead pig	CP002904
GD201008-001	*S. agalactiae*	isolated from a tilapia with meningoencephalitis	NC_018646
ML20171221B6-2	*S. pluranimalium*	isolated from a healthy pig	
1.460	*B. subtilis*	China General Microbiological Culture Collection Center	
WUQT018	*A. hydrophila*	isolated from a healthy porcine tonsil	
WUQT019	*K. pneumoniae*	isolated from a healthy porcine tonsil	
WUQT020	*S. dysgalactiae*	isolated from a healthy porcine tonsil	
WUQT022	*E. coli*	isolated from a healthy porcine tonsil	
WUQT024	*Enterococcus* sp.	isolated from a piglet lung	
WUQT033	*S. hyovaginalis*	isolated from a piglet lung	
ATCC 29213	*S. aureus*	American Type Culture Collection	

**Table 2:** The information on primers used in this study.

Gene	Primer Name	Primer Sequence (5′−3′)	Size (bp)
*E8M05_RS04035*	1−F	GTAGATACTGATGGAGATGGT	594
	1−R	ATAATCGCCTGGTTGAGTC	
*E8M05_RS05155*	2−F	TTGTTCCGTTGTCAGCATA	767
	2−R	AGCACCGATTCTATCCATAA	
*E8M05_RS06300*	3−F	GTTCTGGAATGGTTAGGAATC	409
	3−R	AAGCAGCCGCAATATCAA	
*sodA*	*sodA*−F	ATGGCTATTATTTTACCAAAACTAC	609
	*sodA*−R	TCACTTTGTTGCTTTTGAGTA	

**Table 3:** The results of *S. pasteurianus* identification.

Area	Time	Sample Type	Sample Number	Number of Positive Samples	Positive Rate (%)
Inner Mongolia	2020	cattle feces	6	1	16.67
Chongqing	2021	pig tonsil	50	2	4.00
Yunnan	2021	pig hilar lymph node	50	18	36.00
Jiangsu	2021	pig mesenteric lymph node	50	0	0.00
Guangxi	2021	pig hilar lymph node	45	0	0.00
		pig tonsil	30	0	0.00
Sichuan	2021	pig tonsil	60	3	5.00

**Table 4:** The MICs value and resistance mechanisms.

Classes	Antibiotics	Breakpoints for Resistance (mg/L)	MICs (mg/L)	Resistance Mechanisms
WUSP070				
Macrolides	Erythromycin	≥1	>256	*erm(B)*
Lincosamides	Lincomycin	≥1	>256	*lnu(B)*, *erm(B)*, *lsa(E)*
	Clindamycin	≥1	>256	*lnu(B)*, *lnu(B)*, *lsa(E)*
Tetracyclines	Doxycycline	≥1	32	*tet(O/W/32/O)*, *tet(L)*
Aminoglycosides	Gentamicin	≥16	>256	*aac(6′)-aph(2″)*
WUSP074				
Macrolides	Erythromycin	≥1	>256	*erm(B)*
Lincosamides	Lincomycin	≥1	>256	*lsa(E)*, *lnu(B)*, *erm(B)*
	Clindamycin	≥1	>256	*lsa(E)*, *lnu(B)*, *erm(B)*
Tetracyclines	Doxycycline	≥1	32	*tet(O/W/32/O)*, *tet(L)*, *tet(O)*

## Data Availability

The data supporting this study’s findings are available from the corresponding author upon reasonable request.

## Supplementary Materials

The following supporting information can be downloaded at: https://www.mdpi.com/article/10.3390/pathogens12040615/s1, Table S1. The list of human cases infected by *S. pasteurianus* [38,39,40,41,42,43,44,45,46,47,48,49,50,51,52,53,54,55,56,57]; Table S2. The list of animal cases infected by *S.pasteurianus*; Table S3: The *sodA* sequence identity (from positions 25 to 510) of positive samples with that of type strain NCTC 13784; Table S4: The predicted virulence factors in strains WUSP070 and WUSP074.
