# Supplementary material for: The Identification of Streptococcus pasteurianus Obtained from Six Regions in China by Multiplex PCR Assay and the Characteristics of Pathogenicity and Antimicrobial Resistance of This Zoonotic Pathogen
Source: Pathogens. 2023 Apr 18;12(4):615. doi: 10.3390/pathogens12040615 (PMC10142533; doi:10.3390/pathogens12040615)
Supplement: Supplementary file 1 [file pathogens-12-00615-s001.zip › Table S1-pathogens-2269408.pdf]

Table S1. The list of human cases infected by *S. pasteurianus*

Country	Time of publication	Patient	Symptom	References
America	2000	infant	meningitis	[38]
	2003	neonate	meningitis	[7]
	2010	adult	meningitis	[39]
	2012	neonate	meningitis	[23]
	2013	adult	septicemia	[19]
	2015	twin infants	sepsis and meningitis	[9]
	2016	adult	bacteremia	[15]
	2019	neonate	meningitis, endocarditis, and pneumonitis	[40]
	2021	infant	ductal stent endocarditis	[5]
	2022	infants	bacteremia and/or meningitis	[41]
Argentina	2013	child	meningitis and bacteremia	[42]
Australia	2018	adult	meningitis	[43]
Britain	2010	adult	meningitis	[6]
China	2013	adult	intrauterine infection and post-partum bacteraemia	[8]
		adult	splenic abscess	[11]
	2019	adults	bacteremia, infective endocarditis, meningitis, etc	[26]
	2021	neonates	bacteremia and meningitis	[44]
	2023	neonates	sepsis	[25]
Costa Rica	2021	infant	meningitis	[45]
France	2010	neonates	bloodstream infections	[24]
	2016	neonates	sepsis, necrotizing enterocolitis	[14]
Japan	2009	adult	bacterial peritonitis	[12]
		neonate	meningitis	[46]
	2012	neonate	meningitis	[47]
	2014	infant	meningitis	[48]
		adult	infective endocarditis	[49]
	2018	neonate	meningitis	[50]
	2022	adult	bacteremia	[51]
		adult	acute calculous cholecystitis	[52]
Korea	2015	neonate	bacteremia and meningitis	[53]
Netherlands	2015	adult	meningitis	[54]
Portugal	2023	adult	endocarditis	[55]
Spain	2014	adults	biliary tract	[13]
	2015	adults	urinary infection	[4]
Thailand	2012	neonate	meningitis	[56]

Turkey	2014	neonate	sepsis	[10]
India	2020	adult	bacteremia and infective endocarditis	[57]
