# Supplementary material for: The Identification of Streptococcus pasteurianus Obtained from Six Regions in China by Multiplex PCR Assay and the Characteristics of Pathogenicity and Antimicrobial Resistance of This Zoonotic Pathogen
Source: Pathogens. 2023 Apr 18;12(4):615. doi: 10.3390/pathogens12040615 (PMC10142533; doi:10.3390/pathogens12040615)
Supplement: Supplementary file 1 [file pathogens-12-00615-s001.zip › Table S2-pathogens-2269408.pdf]

Table S2. The list of animal cases infected by *S. pasteurianus*

Country	Time of publication	Animal	Symptoms	References
America	2014	turkey poults	acute septicemia	[19]
Austria	2021	goslings	central nervous symptoms	[17]
Brazil	2022	emperor tamarin	valvular endocarditis and sepsis	[21]
Britain	2008	goslings	septicemia	[16]
China	2013	ducklings	meningitis	[18]
	2022	piglet	meningitis	[22]
Italy	2019	calves	neurological symptoms	[20]
