# Supplementary material for: The Identification of Streptococcus pasteurianus Obtained from Six Regions in China by Multiplex PCR Assay and the Characteristics of Pathogenicity and Antimicrobial Resistance of This Zoonotic Pathogen
Source: Pathogens. 2023 Apr 18;12(4):615. doi: 10.3390/pathogens12040615 (PMC10142533; doi:10.3390/pathogens12040615)
Supplement: Supplementary file 1 [file pathogens-12-00615-s001.zip › Table S3-pathogens-2269408.pdf]

Table S3. The *sodA* sequence identity (from positions 25 to 510) of positive samples with that of type strain NCTC 13784

**Sample**	***sodA* sequence identity (from positions 25 to 510) with that of type strain NCTC 13784**
feces-1	100%
tonsil-54	99.18%
tonsil-82	98.97%
tonsil-KT4	98.93%
tonsil-KT27	98.97%
tonsil-KT35	99.18%
lymphonodus-51	99.14%
lymphonodus-54	99.18%
lymphonodus-57	99.18%
lymphonodus-58	99.18%
lymphonodus-60	99.18%
lymphonodus-62	99.13%
lymphonodus-63	99.18%
lymphonodus-64	98.92%
lymphonodus-71	99.14%
lymphonodus-81	99.13%
lymphonodus-83	98.92%
lymphonodus-86	98.92%
lymphonodus-88	98.94%
lymphonodus-89	99.13%
lymphonodus-90	98.95%
lymphonodus-96	98.93%
lymphonodus-97	99.15%
lymphonodus-100	98.95%
