# Supplementary material for: The Identification of Streptococcus pasteurianus Obtained from Six Regions in China by Multiplex PCR Assay and the Characteristics of Pathogenicity and Antimicrobial Resistance of This Zoonotic Pathogen
Source: Pathogens. 2023 Apr 18;12(4):615. doi: 10.3390/pathogens12040615 (PMC10142533; doi:10.3390/pathogens12040615)
Supplement: Supplementary file 1 [file pathogens-12-00615-s001.zip › Table S4-pathogens-2269408.pdf]

Table S4. The predicted virulence factors in strains WUSP070 and WUSP074

Gene ID	Start	End	Length	Strand	Annotation
WUSP070					
M0P24_RS00375	69330	70343	1014	+	dTDP-glucose 4,6-dehydratase
M0P24_RS03135	604385	605693	1299	+	PssD/Cps14F family polysaccharide biosynthesis glycosyltransferase
M0P24_RS03425	675712	676566	855	+	dTDP-4-dehydrorhamnose 3,5-epimerase family protein
M0P24_RS03440	678853	679659	807	+	phosphopyruvate hydratase
M0P24_RS03445	679659	680870	1212	+	glucose-1-phosphate thymidylyltransferase
M0P24_RS05770	1142023	1142520	498	−	RfbA glycosyltransferase
M0P24_RS05775	1142532	1143008	477	−	dTDP-4-dehydrorhamnose reductase
M0P24_RS05815	1149837	1150745	909	+	trigger factor
M0P24_RS06415	1268062	1269111	1050	−	ABC transporter permease
M0P24_RS06420	1269286	1269879	594	−	UTP--glucose-1-phosphate uridylyltransferase GalU
M0P24_RS06425	1269879	1270748	870	−	type I glyceraldehyde-3-phosphate dehydrogenase
M0P24_RS09160	1805305	1806588	1284	−	ABC transporter ATP-binding protein
M0P24_RS10995	2140907	2141821	915	+	LysR family transcriptional regulator
WUSP074					
M0P28_RS01700	294896	296179	1284	+	dTDP-glucose 4,6-dehydratase
M0P28_RS04060	768445	769314	870	+	GbpC/Spa domain-containing protein
M0P28_RS04065	769314	769907	594	+	dTDP-4-dehydrorhamnose 3,5-

Gene ID	Start	End	Length	Strand	Annotation
					epimerase family protein
M0P28_RS04070	770082	771131	1050	+	phosphopyruvate hydratase
M0P28_RS04195	799732	801387	1656	−	glucose-1-phosphate thymidylyltransferase RfbA
M0P28_RS04645	890694	891602	909	−	dTDP-4-dehydrorhamnose reductase
M0P28_RS07390	1443721	1444527	807	−	trigger factor
M0P28_RS07405	1446814	1447668	855	−	ABC transporter permease
M0P28_RS07950	1555210	1560105	4896	−	UTP--glucose-1-phosphate uridylyltransferase GalU
M0P28_RS08135	1596129	1597427	1299	−	type I glyceraldehyde-3-phosphate dehydrogenase
M0P28_RS10980	2165137	2166150	1014	−	LysR family transcriptional regulator
M0P28_RS11380	2233620	2234534	915	+	NFACT RNA binding domain-containing protein
